# Hybrid improved capuchin search algorithm for plant image thresholding

**DOI:** 10.3389/fpls.2023.1122788

**Published:** 2023-01-26

**Authors:** Shujing Li, Zhangfei Li, Qinghe Li, Mingyu Zhang, Linguo Li

**Affiliations:** ^1^ School of Computer and Information Engineering, Fuyang Normal University, Fuyang, China; ^2^ School of Computer, Nanjing University of Posts and Telecommunications, Nanjing, China

**Keywords:** capuchin search algorithm, chaotic mapping, opposite-based learning, levy flight, plant image thresholding

## Abstract

With the development and wider application of meta-heuristic optimization algorithms, researchers increasingly apply them to threshold optimization of multi-level image segmentation. This paper explores the performance and effects of Capuchin Search Algorithm (CAPSA) in threshold optimization. To solve problems of uneven distribution in the initial population of Capuchin Search Algorithm, low levels of global search performance and premature falling into local optima, this paper proposes an improved Capuchin Search Algorithm (ICAPSA) through a multi-strategy approach. ICAPSA uses chaotic opposite-based learning strategy to initialize the positions of individual capuchins, and improve the quality of the initial population. In the iterative position updating process, Levy Flight disturbance strategy is introduced to balance the global optimization and local exploitation of the algorithm. Finally, taking Kapur as the objective function, this paper applies ICAPSA to multi-level thresholding in the plant images, and compares its segmentation effects with the original CAPSA, the Fuzzy Artificial Bee Colony algorithm (FABC), the Differential Coyote Optimization Algorithm (DCOA), the Modified Whale Optimization Algorithm (MWOA) and Improved Satin Bowerbird Optimization Algorithm (ISBO). Through comparison, it is found that ICAPSA demonstrates superior segmentation effect, both in the visual effects of image segmentation and in data comparison.

## 1 Introduction

Due to the advantages of fast convergence rate and high accuracy of meta- heuristic optimization algorithms, many researchers increasingly apply them to real-world problems, to improve the application effects of computer-aided design in the engineering field (Pare et al., 2020). Ma et al. (2011) combine Artificial Bee Colony algorithm (ABC) with image segmentation to improve the segmentation accuracy of Synthetic Aperture Radar (SAR) images. Hemasian-Etefagh and Safi-Esfahani (2019) apply Whale Optimization Algorithm (WOA) to solve the scheduling problem of cloud computing, thus reducing the execution and response time of scheduling tasks, and increasing the computing throughput in the context of a cloud environment. (Gholizadeh and Baghchevan, 2017) apply Firefly Algorithm (FA) in the seismic design of steel frames to locate the optimal goal more quickly. Specifically, in the field of plant image processing, with the change of environment and the development of information technology, many countries regard ecological construction and the development of smart agriculture as national strategies. In order to improve the benefits of ecological environment and agricultural development, scholars have deeply integrated traditional plant planting technology with the internet of things, 5G and artificial intelligence technology (Maray et al., 2022; Ruwona and Scherm, 2022). This development model reduces the labor of workers and improves production efficiency. However, in the current development process, the main problem encountered is that the frequent occurrence of diseases has led to reduction of output or even crop failure. In order to reduce the loss of plant quality and economy caused by diseases, targeted screening and diagnosis in advance are required during plant growth (Patel et al., 2023). The traditional way is manual identification, relying on experience, high cost and low accuracy. In recent years, the recognition based on computational image processing is more efficient and accurate, the processing steps include: image preprocessing, image segmentation, feature extraction and recognition, and the higher accuracy of image segmentation, the higher accuracy of recognition (Xue et al., 2021 **;** Hasan et al., 2022). However, segmentation accuracy and efficiency directly affect the application of segmentation technology in plant applications. Making use of the advantages of meta-heuristic algorithms in multi-level image thresholding is thus considered an effective plant disease assisted treatment (Li et al., 2022).

As one of the main technical means of image segmentation, multi-level thresholding based on specific objective function is fused with a meta-heuristic algorithm, and has been thus used successfully (Merzban and Elbayoumi, 2019; Xue et al., 2022). Image segmentation divides an image into several regions according to characteristics such as texture, color, brightness, contrast, shape, and size (Pare et al., 2020; Rodríguez-Esparza et al., 2020; Xue et al., 2019). Specifically, thresholding segmentation is divided into bi-level (Bao et al., 2019) and multi-level thresholding (Ma and Yue, 2022). Bi-level is used mainly for image binarization, and its application field is limited. The multi-level can dynamically improve segmentation accuracy in response to actual needs by adjusting the number of thresholds, although such increase of thresholds leads to an explosive increase in computational complexity. Therefore, a meta-heuristic algorithm based on specific objective functions can effectively strike the balance between these problems (Ma and Yue, 2022). The common objective functions include Kapur entropy (Upadhyay and Chhabra, 2020), Minimum Cross Entropy (MCE) (Wang and Song, 2022), Tsallis (Lin et al., 2020).

Regarding the selection of objective functions, Sathya et al. (2021) compare and analyze the segmentation effect of Exchange Market Algorithm (EMA) using Kapur, Otsu and MCE. Through comparison of visual effects and quantitative data after standard image segmentation, they conclude that the Kapur-based method has faster processing speed and better segmentation effect. Li et al. (2016) taking Kapur as the objective function, prove the efficiency of the fuzzy ABC (FABC) in multi-level image thresholding through experimental comparison using BSD500 dataset. Kalyani et al. (2021) use EMA algorithm based on Kapur and MCE to segment three different images, comparing it with Krill Herd (KH), Teaching-Learning based Optimization (TLBO) and Cuckoo Search Algorithm (CSA). They find that the Kapur-based algorithm has faster convergence speed and segmentation accuracy, and thus has better practical application value. Rajinikanth et al. (2021) improve Moth Flame Optimization (MFO) algorithm and compare the image thresholding segmentation effects based on Kapur and Tsallis. Through comparison of multiple experimental results, they find this method achieves better segmentation effect than other methods of the same kind. Chen et al. (2022) taking Kapur as the objective function, integrate the multi-strategy driven Shuffled Frog-Leaping Algorithm with Horizontal and Vertical Crossover search (HVSFLA) to perform multi-level thresholding, achieving remarkable segmentation effect.

Compared with traditional methods (Kittler and Illingworth, 1986; Otsu, 1979), meta-heuristic optimization algorithms perform more efficiently in multi-level image thresholding. Zhao et al. (2021) use random spare strategy and logistic chaos enhancement strategy to optimize Ant Colony Optimization (ACO), effectively improving the convergence speed and accuracy of ACO and enhancing the ability of the algorithm to evade local optima. The experimental results show that the improved ACO algorithm is satisfactory. To optimize the multi-level thresholding of gray-scale images, Abdel-Basset et al. (2022) combine Linearly Convergence Increasing and Local Minima Avoidance Technique (LCMA) and Ranking-based Update Method (RUM) to improve the Wheel Optimizer Algorithm (WOA). The former moves the individual at the worst position in the population into the range of the best current scheme to avoid local optimization. The latter replaces the unfavorable solution with a better one. Similarly, based on Kapur, 13 test images of Berkeley segmentation dataset BSD are verified, demonstrating that the improved WOA is superior to other methods in both segmentation image quality and convergence speed. Ewees et al. (2020) propose an Improved Artificial Bee Colony Algorithm integrating the Sine Cosine Algorithm (ABCSCA). This algorithm uses ABC to narrow the search scope and optimize the threshold, while SCA can determine the global optimal threshold and obtain the optimal solution. To measure the effects of ABCSCA, Otsu and fuzzy entropy are used as the objective functions to segment 19 images. Compared with the original ABC, SCA algorithms and Hybrid Swarm Optimization (FASSO), the algorithm has more obvious advantages in image segmentation effect, and convergence speed. Anitha et al. (2021) improve Modified Whale Optimization Algorithm (MWOA) by using cosine function, and obtain better image segmentation quality and convergence speed than PSO, ABC and other algorithms using Otsu as the objective function. Li et al. (2021) use the differential evolution strategy to improve the population updating mechanism of Coyote Optimization Algorithm (DCOA), not only improving the convergence speed of the algorithm, but also its image segmentation accuracy, rendering it superior to standard COA, Gray Wolf Optimizer (GWO), modified Discrete Gray Wolf Optimizer (DGWO) and other methods. To solve the problem of identifying corn pests and diseases, Chen et al. (2021) improve Particle Swarm Optimizer (PSO) with an elite based advantage scheme to form an Enhanced Comprehensive Learning Particle Swarm Optimizer (ECLPSO). Compared with the Comprehensive Learning Particle Swarm Optimizer (CLPSO) and Hybridizing Sine Cosine Algorithm with Differential Evolution (SCADE) algorithm in the corn leaf disease image in the public database of a plant village company, the results show this method to be superior to other comparison algorithms in locating the best threshold, and have higher convergence accuracy. Li et al. (2022) proposed a strategy based on chaos initialization and Cauchy mutation to improve Satin Bowerbird Optimization Algorithm (SBO), and verified its values in Kaggle plant image dataset. The comparison between the fuzzy Modified Discrete Grey Wolf Optimizer with aggregation strategy (FMGWO) and the fuzzy Coyote Optimization Algorithm (FCOA) proves that the improved ISBO has higher accuracy in the field of plant image segmentation.

Meta-heuristic optimization algorithms can effectively improve the computational efficiency in multi-level image thresholding. However, there is room for further improvement in population initialization and global search ability (Pare et al., 2020; Li et al., 2022). Therefore, this paper seeks to improve CAPSA, comparing its efficacy in plant image segmentation with other algorithms. CAPSA is a novel meta-heuristic optimization algorithm proposed by Braik et al. (2021) in 2021. This algorithm divides the population into two groups with distinct functions so as to strike the balance between global search ability and local exploitation ability. Compared with other similar algorithms, it has higher convergence speed and accuracy, but in later iterations, CAPSA is also prone to fall into local optima.

Therefore, this paper uses Tent chaotic iterative mapping and Opposite-based learning strategy to initialize the population, improve the quality of the initial population, make its distribution more uniform, thus eschewing the premature local optima of CAPSA. In the position updating strategy of the algorithm, Levy Flight strategy is integrated to balance the ability of global search and local exploitation of the algorithm to form an Improved Capuchin Search Algorithm (ICAPSA). To verify the effects of ICAPSA, this paper uses Kapur entropy as the objective function to segment plant images into multiple thresholds, and compares the experimental results with the results of the FABC (Li et al., 2016), MWOA (Anitha et al., 2021), DCOA (Li et al., 2021) et al.

The remainder of this paper is as follows: In the second section, the original CAPSA and its model construction is described. In the third section, the improved strategy of the algorithm is presented in detail. In the fourth section, to verify the practical effect of ICAPSA in plant image segmentation, six plant images are selected for visual and quantitative data comparative analysis. Finally, the fifth section concludes.

## 2 Description and model construction of Capuchin Search Algorithm (CAPSA)

### 2.1 Original CAPSA description

Capuchin search algorithm (CAPSA) is a new algorithm that simulates the foraging behavior of capuchin populations in Brazil and South America. Each population includes about 10 to 35 capuchins, and each population has an alpha (α) monkey commanding this group, called the leader, who is responsible for finding food sources for this group. The remaining capuchins are called followers. If the alpha monkey cannot obtain sufficient food sources in time, the group will be divided into smaller sub-groups to forage independently.

Capuchin monkeys use jumping, swinging and climbing in the process of foraging for food. Jumping allows capuchin monkeys to have a wider search range. Swinging and climbing are used to improve local search ability. Followers will update their positions according to the leader’s position and their own positions, and finally improve the foraging rate and success rate.

### 2.2 Initialization of CAPSA

Like other similar algorithms, capuchin search algorithm is also a population-based search algorithm. It randomly initializes the population. Each individual of the population represents the candidate scheme of the target problem. The initialized individuals are divided into two categories: alpha monkeys and followers.

Assume that the capuchin monkey population has *n* individuals, and the search space is d-dimensional. The initial position can then be expressed by the following matrix:


(1) $$\text{x}=\left[\begin{array}{ccccc} {\text{x}}_{1}^{1} & {\text{x}}_{2}^{1} & \cdots & \cdots & {\text{x}}_{\text{d}}^{1}\\ {\text{x}}_{1}^{2} & {\text{x}}_{2}^{2} & \cdots & \cdots & {\text{x}}_{\text{d}}^{2}\\ \vdots & \vdots & \vdots & \vdots & \vdots \\ {\text{x}}_{1}^{\text{n}} & {\text{x}}_{2}^{\text{n}} & \cdots & \cdots & {\text{x}}_{\text{d}}^{n} \end{array}\right]$$

Where *x* represents the positions of capuchin monkeys, *n* the number of capuchins, *d* the dimension of the problem, and $x_{\text{d}}^{i}$ the position of the i -th capuchin monkey in d-dimensional space.

Initialize the position of each capuchin individual by (2):


(2) $$x^{\text{i}}=ub_{j}+r\times (ub_{j}-lb_{j})$$

Where ub_j_ and lb_j_ represent the upper and lower bounds of the capuchin monkey in the dimensional space respectively, and *r* is a random number uniformly generated inside [0,1].

### 2.3 Evolution of CAPSA

In Capuchin search algorithm, the updating of capuchin positions depends on their current position and best position along with the location of food *F*. *F* is the target of capuchin monkeys in d-dimensional search space. The position of the leader and those of its followers relative to where food *F* is, are updated as follow steps.

Jump on the tree: the leader (α monkey) can jump from tree to tree or from the current branch to other branches of the same tree, then α monkey’s position updating formula is as follows.


(3) xji=Fj+Pbf(vji)2sin(2θ)g,i<n2,0.1<ϵ≤0.20

Where $x_{\text{j}}^{i}$ represents the position of α monkey and its followers in the j dimension, F_j_ the position of food in the j-th dimension, *∈* the random number generated inside [0, 1], P_bf_ is the probability that the tail provides balance in the capuchin jumping process, g the gravitational acceleration, g=9.81, θ is the capuchin jumping angle, τ represents the life cycle, systematically decreases in the whole iteration process, $v_{\text{j}}^{i}$ represents the speed of the i-th capuchin in the j-th dimension.

The jumping angle of a capuchin monkey can be defined by (4):


(4) $$\text{θ}=\frac{3}{2}\text{r}$$

Where r is a random number generated uniformly in the range [0,1]. To balance the global search ability and local exploitation ability, CAPSA introduces the concept of life cycle τ as shown in (5):


(5) $$\text{τ}={\text{β}}_{0}\text{e}-{\text{β}}_{1}{\left(\frac{k}{\text{K}}\right)}^{{\text{β}}_{2}}$$

Where k and K respectively represents the current iteration number and the maximum iteration number. The values of parameters β_0_, β_1_ and β_2_ are respectively 2, 21 and 2. The exponential function has a great impact on updating the positions of Capuchin monkeys, the exploration and development of regions, and the quick locating of food sources.

The velocity of the i-th capuchin monkey in the j dimension is shown in (6):


(6) $${\text{v}}_{\text{j}}^{i}={\text{ρv}}_{j}^{\text{i}}+{\text{τa}}_{1}{\text{(x}}_{{\text{best}}_{\text{j}}}^{\text{i}}-x_{\text{j}}^{i}){\text{r}}_{1}+{\text{τa}}_{2}{\text{(F}}_{\text{j}}-{\text{x}}_{j}^{\text{i}}){\text{r}}_{2}$$

Where i=1, 2, 3, …,n, j stands for the dimension of the problem, ${\text{v}}_{j}^{\text{i}}$ the current velocity of the i-th capuchin monkey in the j dimension, ${\text{x}}_{j}^{\text{i}}$ the current position of the i-th capuchin monkey in the j-th dimension. ${\text{x}}_{{\text{best}}_{\text{j}}}^{\text{i}}$ the best velocity of the i-th capuchin monkey in the j-th dimension, F_j_ the position of food in the j-th dimension. a_1_ and a_2_ are two normal numbers, their values can be taken at 1 or 0, representing the impact of ${\text{x}}_{{\text{best}}_{\text{j}}}^{\text{i}}$ and F_j_ on the velocity of capuchins. r_1_ and r_2_ are random numbers generated evenly in the range [0, 1]. ρ is the inertia coefficient with a value of 0.7, indicating the impact of the current velocity on the motion.

Jumping on the ground: Capuchins can jump from one place to another on the ground, from one side of the riverbank to the other, or wander normally to search for food. In this case, the position updating formula of α monkey and its followers is as follows:


$${\text{x}}_{j}^{\text{i}}={\text{F}}_{\text{j}}+\frac{{\text{P}}_{\text{ef}}{\text{P}}_{\text{bf}}{\left({\text{v}}_{j}^{\text{i}}\right)}^{2}\sin(2\text{θ})}{\text{g}},$$

(7) i<n2,0.2<ϵ≤0.30

Where P_ef_ represents the elasticity probability of a capuchin monkey moving on the ground, θ is defined in (4).

On the other hand, when α monkey wanders normally, the position updating can be shown in (8):


(8) xji=xji+vji, i<n2,0.3<ϵ≤0.50

From these two jumping mechanisms, it can see that capuchin monkeys have two basic parameters in the process of approaching food. the probability P_bf_ that the tail provides balance in the process of jumping, and the elasticity probability P_ef_ of moving on the ground. These two coefficients balance their global search and local exploitation ability, and their values are taken at 0.7 and 9, respectively.

Swing: some α monkeys and their followers will swing their bodies over the branches with their tails and perform local exploitation to forage food. The positions of capuchin monkeys are updated as follows:


(9) xji=Fj+τPbf×sin(2θ), i<n2,0.5<ϵ≤0.75

Where θ is defined in (4);

Climbing: in the process of foraging, some α monkeys and their followers will climb up a tree or branches, and then climb down. This behavior is also local exploitation. Their positions are as follows:


(10) xji=Fj+τPbf(vji−vj−1i), i<n2, 0.75<ϵ≤1.0

Where ${\text{v}}_{j}^{\text{i}}$ is the current velocity of the i-th capuchin in the j-th dimension, ${\text{v}}_{\text{j}-1}^{\text{i}}$ is the previous velocity of the i-th capuchin in the j-th dimension.

Random migration of capuchin monkeys: in foraging food, capuchin monkeys will randomly search in several new directions to effectively explore the forest to search for better food sources. Random migration is shown in (11):


(11) xji=τ×[lbj+ϵ×(ubj−lbj)], i<n2,ϵ<Pr

Where Pr is a normal number with a value of 0.1, representing the probability of capuchin monkeys performing random search. ub_j_ and lb_j_ are the upper and lower bounds of the j-th dimensional search space, respectively. The random migration of capuchins not only enhances the global search ability, but also prevents CAPSA from falling into local optima.

To sum up, as is shown in (3) to (10), the capuchins will change their positions depending on the availability of food, their search target. This situation is particularly apparent when r > 0.1.

On the other hand, when r≤0.1, capuchin monkeys are more likely to randomly change their positions in the search domain so as to explore different areas for food. In this case, parameter τ can enhance the search space available for exploration.

The positions of the followers are updated according to the position of α monkey, as is shown by (12).


(12) $${\text{x}}_{\text{f}}={\text{x}}_{\text{i}}+{\text{v}}_{0}\text{t}+\frac{1}{2}{\text{at}}^{2}$$

Where x_f_ is the final displacement, x_1_ is the initial displacement. t is the time, v_0_ represents the initial velocity. a is the acceleration, its value is shown in (13):


(13) $$\text{a}=\frac{\Delta \text{v}}{\Delta \text{t}}=\frac{{\text{v}}_{\text{f}}-{\text{v}}_{0}}{{\text{t}}_{1}-{\text{t}}_{0}}$$

Where t_1_ and t_0_ represent the last time and the first time respectively. The parameter v_f_ represents the final velocity, its value is shown in (14):


(14) $${\text{v}}_{\text{f}}=\frac{\Delta \text{x}}{\Delta \text{t}}=\frac{{\text{x}}_{\text{f}}-{\text{x}}_{0}}{{\text{t}}_{1}-{\text{t}}_{0}}$$

Substitute (14) into (13), and set the initial velocity v_0_=0, then a can be shown by (15):


(15) $$\text{a}=\frac{{\text{x}}_{\text{f}}-{\text{x}}_{0}}{{\left({\text{t}}_{1}-{\text{t}}_{0}\right)}^{2}}$$

In optimization, t_1_ represents iteration, t_1_-t_i-1_ represents the difference between successive iterations, equal to a value of 1. Based on (12) and (15), (16) represents the position updating formula of the followers:


(16) $${\text{x}}_{j}^{\text{i}}=\frac{1}{2}({\text{x}}_{j}^{\prime \text{i}}+{\text{x}}_{j}^{\text{i}-1}),\text{ n}/2\leq \text{i}\leq \text{n}$$

Where ${\text{x}}_{j}^{\text{i}}$ represents the current position of the followers in the j-th dimension. ${\text{x}}_{j}^{\text{i}-1}$ represents the previous position of the followers in j-1-th dimension. ${\text{x}}_{j}^{\prime \text{i}}$ the current position of the leader in the j-th dimension.

## 3 The Hybrid Improved CAPSA

### 3.1 Tent chaotic mapping

**Figure 1:**
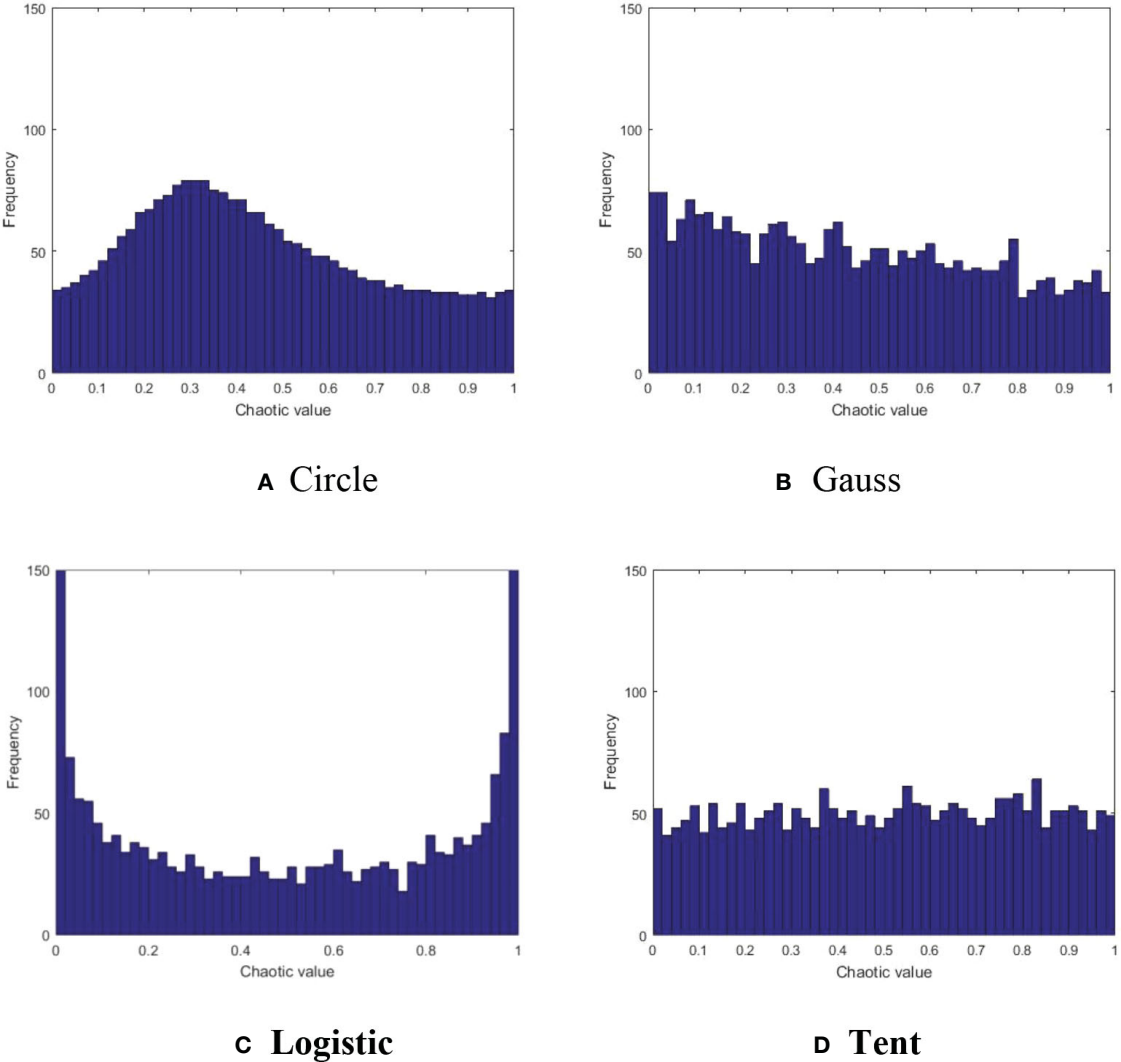
Comparison of four chaotic maps. **(A)** Circle **(B)** Gauss **(C)** Logistic **(D)** Tent.


The Capuchin search algorithm performs well in convergence speed, and also considers the balance between global search and local exploitation. However, similar to other meta-heuristic optimization algorithms, there exists a certain probability of “local optimal” in later iterations, which is attributable to the population random initialization strategy of meta-heuristic optimization algorithm. In constructing the model in section 2.2, it is described that the population initialization of CAPSA is completed randomly according to (2), and the distribution of individual positions of the population is inherently random. As chaotic mapping has the advantages of ergodicity and universality, many researchers use it to optimize the population initialization of meta-heuristic optimization algorithms. Commonly used chaotic map methods include Circle map (Ewees and Abd Elaziz, 2020), Gauss map (Elaziz et al., 2021), Logistic map (Prasad et al., 2021) and Tent map (Li et al., 2020), as shown in (**Figures 1A-D**). Circle chaotic map and Logistic chaotic map have the characteristics of small humps and peaks at both ends, which may cause group aggregation and are not conducive to global search. Compared with Gauss chaotic map, Tent chaotic map has the advantages of more uniform distribution and smaller peak value, which will not affect the convergence speed of the algorithm. Therefore, Tent chaotic map model is selected in this paper. Tent chaotic map model is as follows:

(17) $${\text{x}}_{\text{i}+1}=\{\begin{array}{c} {\text{ x}}_{\text{i}}/\text{β}\;\;\;\;\;\;\;\;\;\;\;\;\;{\text{x}}_{\text{i}}\in (0,\text{β}]\\ (1-{\text{x}}_{\text{i}})/(1-\text{β)}\;{\text{ x}}_{\text{i}}\in (\text{β},1]\; \end{array}$$

### 3.2 Opposite-based learning (OBL)

Sihwail et al. (2020) use Opposite-based learning (OBL) to improve meta-heuristic algorithms, proving the ability of OBL to prevent the algorithm falling into local optima. Relying on chaotic reflection, and the initial population individuals, OBL compares the fitness values of the individuals before and after reflection and adds the higher fitness values to the initial population. It improves the quality and diversity of the population. The steps of OBL are as follows.

Assume that the initial population generated by Tent chaotic map is x_i,j_,(i=1,2,3,…,N;j=1,2,3,…,d) , first, sequence the individuals in the current population, select the optimal value as the elite individual ${\text{x}}_{\text{i},\text{j}}^{\text{e}}=\left({\text{x}}_{\text{i},1}^{\text{e}},{\text{x}}_{\text{i},2}^{\text{e}},{\text{x}}_{\text{i},3}^{\text{e}},\ldots ,{\text{x}}_{\text{i},\text{d}}^{\text{e}}\right)$ , generate the chaotic elite inverse solution $\bar{{\text{x}}_{\text{i},\text{j}}^{\text{e}}}=\left(\bar{{\text{x}}_{\text{i},1}^{\text{e}}},\bar{{\text{x}}_{\text{i},2}^{\text{e}}},\ldots ,\bar{{\text{x}}_{\text{i},\text{d}}^{\text{e}}}\right)$ according to (17), and then deal with the points beyond the boundary according to (18).


(18) $$\bar{{\text{x}}_{\text{i},\text{j}}^{\text{e}}}=\text{k}\times ({\text{α}}_{\text{j}}+{\text{β}}_{\text{j}})-{\text{x}}_{\text{i},\text{j}}^{\text{e}}$$

Where k∈(0,1) is the inverse coefficient, α_j_ the minimum value of the feasible solution, β_j_ the maximum value of the feasible solution. Finally, the initial solution generated by chaotic map and the solution generated by OBL are sequenced, and the best first *N* individuals are selected as the initial of the population.

### 3.3 Position updating

From the model construction in section 2.2, it knows that when the leader α monkey finds the location of the best food, that is, the optimal solution of the problem. Other followers in the population will follow the leader to approach the location of the best food, indicating that the value will increasingly converge to the “optimal value”. However, this optimal value is not guaranteed to be the optimal one of the whole search spaces. If the algorithm falls into local optimization, the optimization range will be unexpectedly narrowed, and optimization accuracy undermined. In order to reduce the probability of Capuchin algorithm falling into local optima in iteration, this paper introduces Levy Flight disturbance strategy (He et al., 2023) to disturb the population when updating the position of local exploitation, so that the global search and local exploitation can be balanced. As far as the application of ICAPSA is concerned, Levy Flight is integrated into Jump on the tree [formula (3)], Jumping on the ground [formula (7)-(8)], Swing [formula (9)], Climbing [formula (10)] and Random migration of capuchin monkeys [formula (11)], as described in the Pseudo code of section 3.4. In detail, Levy Flight runs through all stages of population position updating.

Levy Flight is a random walk strategy that conforms to Levy distribution. It is a strategy proposed by academics according to the foraging process of natural organisms. As it has the characteristics of long-distance and short-distance staggering motion and fully random direction, researchers often use it to optimize meta-heuristic algorithms, improve the global search range of the algorithm, and evade falling into the trap of local optima. Its mathematical model is shown in (19):


(19) $$\text{levy(α})=0.05\times \frac{\text{x}}{{\left|\text{y}\right|}^{1/\text{α}}}$$

Where *x* and *y* are two normally distributed variables subject to standard deviation σ_x_ and σ_y_, the calculation formula is as follows:


(20) $$\text{x}=\text{Normal(}0,\sigma_{\text{x}}^{2})$$

(21) $$\text{y}=\text{Normal(}0,\sigma_{\text{y}}^{2})$$

(22) $${\text{σ}}_{\text{x}}={\left[\frac{\text{Γ(}1+\text{α})\text{sin(}\frac{\text{πα}}{2})}{\text{Γ(}\frac{1+\text{α}}{2})\text{α}2^{\frac{\left(\text{α}-1\right)}{2}}}\right]}^{1/\text{α}},{\text{σ}}_{\text{y}}=1,\text{α}=1.5$$

### 3.4 Pseudo code of ICAPSA

After setting the initial parameter*∈* and *P*, Tent Chaotic Mapping and OBL are used to optimize the positions of *N* capuchins. Tent Chaotic Mapping is used to ensure that the initial population has a higher randomness, and OBL strategy can improve the dispersion of the population, which to some extent reduces the risk of the algorithm falling into the local optimum. To balance the global search and local exploitation, Levy Flight is merged in all stages of ICAPSA’s population position updating, as show in step 15, step 17, step 19, step 22, step 24 and step 27 of ICAPSA’s pseudo code.

**Table:** 

Pseudo code: ICAPSA
1: Initialization parameter*∈* is a random number inside the range [0,1]. 2: Initialization probability parameter *P*=0.5. 3: Initialize the positions of N capuchins with formulas (17) and (18). 4: Calculate the fitness value of each capuchin position. 5: Initialize the velocity of capuchin monkey. 6: Capuchins smaller than *n/2* are randomly selected as leaders and companions, and the remaining capuchins follow the leader. 7: while *t<maxitet* 8: Update the parameter life cycle according to formula (5). 9: For k=1: noP (noP is the number of Capuchins in the population) 10: if (*k*<*n*/2) 11: Use formula (6) to update the velocity of the leader. 12: if (*∈ ≥* 0.1) 13: if (*∈ ≥ P*) 14: if (*∈≤* 0.2) 15: Update the position of the leader jumping on the tree with formula (3) and (19). 16: else if (0.2<*∈ ≥* 0.30) 17: Update the position of the leader who jumps the riverbank with formula (7) and (19). 18: else 19: Update the position of the leader wandering on the ground with formula (8) and (19). 20: end if 21: else if (0.5<*∈ ≤* 0.75) 22: Update the position of the leader swinging between the branches with formula (9) and (19). 23: else if (0.75<*∈ ≥* 1.0) 24: Update the position of the leader climbing the tree with formula (10) and (19). 25: end if 26: else 27: Update the position of the leader of the random migration with formula (11) and (19). 28: end if 29: else 30: Update the positions of the followers with formula (16). 31: end if 32: end for 33: Calculate the fitness value of each individual. 34: end while 35: Obtain the best solution

## 4 Analysis and comparison of experimental results

### 4.1 Parameter setting and discussion

**Figure 2:**
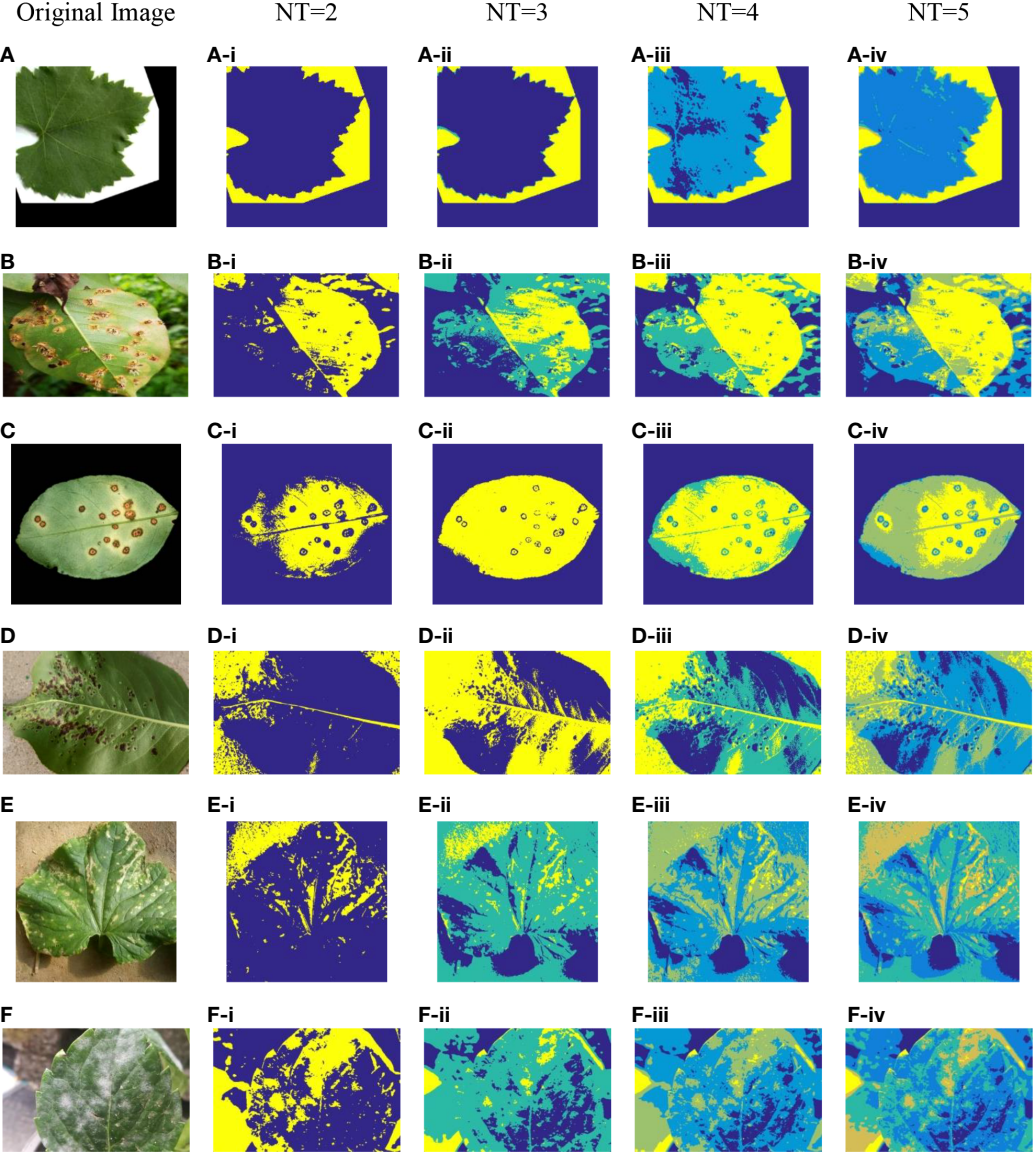
Plant image segmentation based on CAPSA. **(A)** Leaf01. **(B)** Apple Brown Spot. **(C)** Citrus Bacterial Canker. **(D)** Black Spot. **(E)** Bacterial Keratosis of Cucumber. **(F)** Powdery Mildew.

To demonstrate the efficacy of the improved capuchin search algorithm in plant image thresholding, this paper selects six plant images (**Figures 2A-F**) in the Kaggle plant image dataset (https://www.kaggle.com/datasets/asheniranga/leaf-disease-dataset-combination, https://www.kaggle.com/datasets/vipoooool/new-plant-diseases-dataset) for verification. In addition to comparing the results with the original capuchin search algorithm, the results are also fully compared and analyzed with FABC (Li et al., 2016), MWOA (Anitha et al., 2021), DCOA (Li et al., 2021) et al. To reflect the best performance of ICAPSA, the parameter setting is discussed first. In this paper, the number of thresholds (NT) is set to 2, 3, 4, 5. the objective function is set as the Kapur which commonly used in thresholding image segmentation (Li et al., 2022), and the parameter selection feature similarity FSIM (Pare et al., 2020) and peak signal-to-noise ratio PSNR (Abualigah et al., 2022) are compared. The experiments performed in our work are run on Windows10-64bit, Intel processor and 16GB running memory and the programming software is Matlab 2016a. The initial experimental parameter values are shown in **Table 1**.

**Table 1:** Experimental related parameter setting.

Parameter	noP	NT	maxiter
Value	40	2,3,4,5	500

In **Table 1**, *noP* represents the number of capuchins, *NT* represents the number of thresholds, *maxiter* represents the maximum number of iterations. Next, we discuss the rationality optimization of parameter selection in **Table 1** for plant image threshliding.

To reflect the impact of a single variable *noP* on the experimental results, the threshold number *NT* is set to 5 and the *maxiter* is set to 500. Then, by setting the number of individuals in different populations, the image threshold segmentation of the Leaf01 plant image is performed, and the PSNR value is recorded. The average value is calculated through multiple experiments as shown in **Table 2**. It can be observed that the best experimental results are achieved when the population individual number *noP* is set to 30.

**Table 2:** Effect of experimental results by noP.

noP	10	20	30	40	50	60
PSNR	26.5038	26.7058	**26.7737**	26.6898	26.7129	26.5218
FSIM	0.8513	0.8579	**0.8624**	0.8577	0.8541	0.8508

On the premise that the number of individuals *noP* of Capuchin monkey population is set to 30, to determine the impact of the maximum number of iterations on the segmentation effect, **Table 3** lists the maximum PSNR and FSIM values of Leaf01 image through numerous experiments. As can be seen, when the maximum number of iterations is set to 200, the segmentation effect is the best.

**Table 3:** Effect of experimental results by maximum iteration number (maxiter).

maxiter	50	100	200	300	400	500
PSNR	25.1092	26.5741	**26.8305**	26.2416	26.6439	26.5458
FSIM	0.8329	0.8385	**0.8583**	0.8136	0.8583	0.8577

### 4.2 Plant image segmentation results with ICAPSA

**Figure 3:**
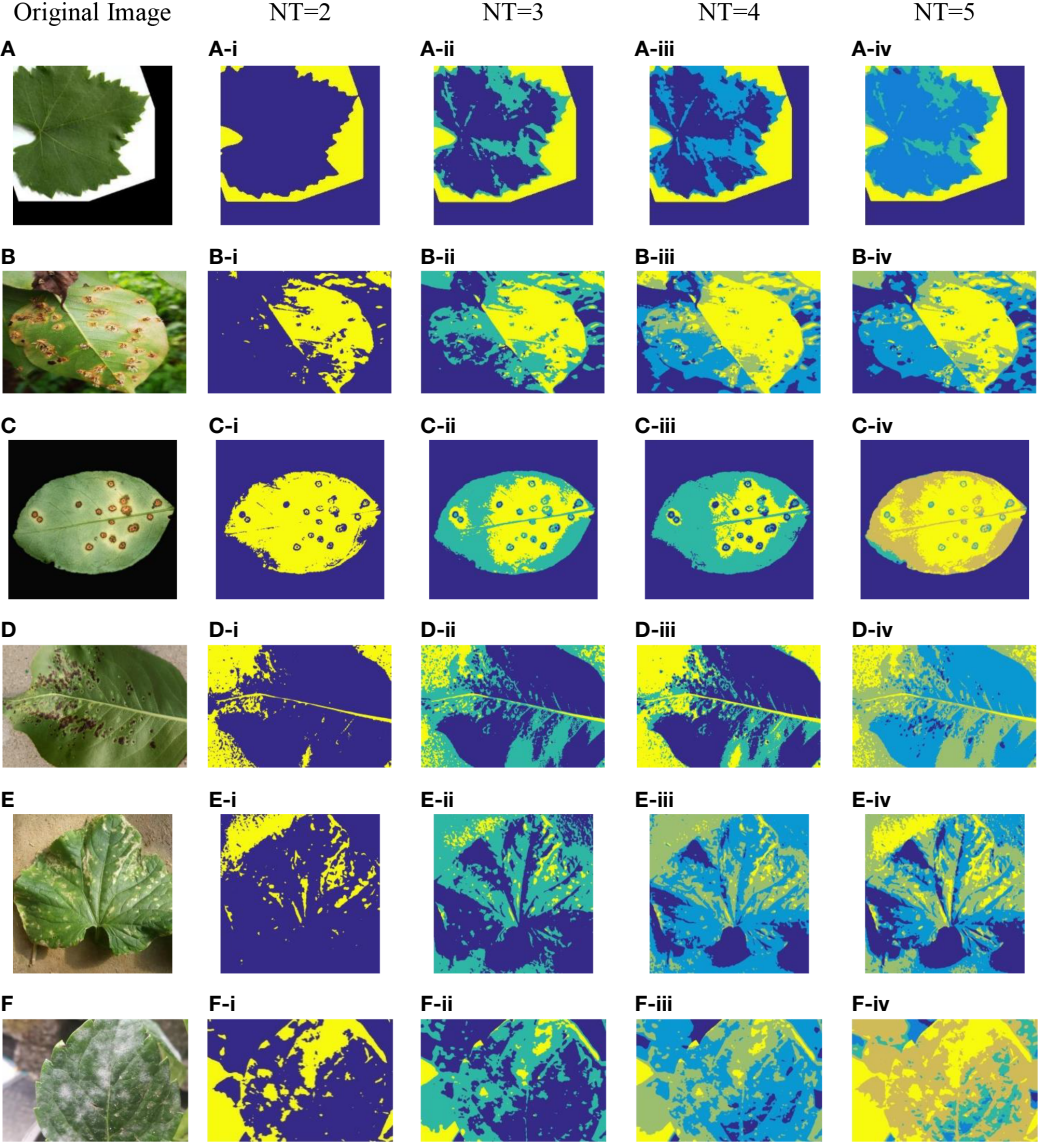
Plant image segmentation based on ICAPSA. **(A)** Leaf01. **(B)** Apple Brown Spot. **(C)** Citrus Bacterial Canker. **(D)** Black Spot. **(E)** Bacterial Keratosis of Cucumber. **(F)** Powdery Mildew.

To reflect the threshold segmentation efficacy of ICAPSA for different plant images, six plant images are selected, with Kapur as the objective function to obtain the optimal segmentation solution under different threshold numbers. **Figures 2**, **3** show the visual diagram of segmentation effect based on CAPSA and ICAPSA under different thresholds. **Table 4** demonstrates the segmentation quality evaluation results (PSNR and FSIM values) of ICAPSA with different thresholds.

**Table 4:** Experimental results of ICAPSA with different threshold numbers.

Image	NT	Thresholds	PSNR	FSIM
**Leaf01**	2 3 4 5	141 213 21 155 225 51 119 190 224 5 61 130 159 233	16.2911 22.5087 25.7779 26.8305	0.7229 0.7858 0.8015 0.8583
**Apple Brown Spot**	2 3 4 5	147 221 63 137 207 33 82 138 180 27 116 156 192 252	18.6521 21.2129 22.7506 23.9403	0.6066 0.6904 0.7467 0.7953
**Citrus Bacterial Canker**	2 3 4 5	44 204 60 168 232 79 142 200 254 13 36 58 122 168	21.3084 24.4853 26.0432 27.1299	0.7655 0.8379 0.8805 0.8943
**Black Spot**	2 3 4 5	98 211 97 162 245 49 165 218 248 17 67 134 202 243	21.5449 23.7496 25.1650 26.2449	0.6556 0.7590 0.8137 0.8436
**Bacterial keratosis of cucumber**	2 3 4 5	134 231 87 167 214 28 92 146 246 36 98 140 204 255	19.2370 21.8199 23.6029 24.6088	0.6028 0.7081 0.7808 0.8402
**Powdery Mildew**	2 3 4 5	90 205 94 159 220 42 111 153 236 18 58 78 104 200	20.0592 21.8992 23.2750 24.9361	0.5600 0.6061 0.6390 0.7018

In **Figures 2**, **3**, the segmentation effects are shown when *NT* was set to 2(**Figure 2** from (A-i) to (F-i) and **Figure 3** from (A-i) to (F-i)), 3(**Figure 2** from (A-ii) to (F-ii) and **Figure 3** from (A-ii) to (F-ii)), 4(**Figure 2** from (A-iii) to (F-iii) and **Figure 3** from (A-iii) to (F-iii)) and 5 (**Figure 2** from (A-iv) to (F-iv) and **Figure 3** from (A-iv) to (F-iv)) respectively based on original CAPSA and ICAPSA. As can be observed, when *NT* was 2, the background and disease areas were better segmented and located. When *NT* was set to 3, 4 and 5, the segmentation effect was gradually refined, and the regional details were better defined. But in comparison with CAPSA and other algorithms, it is difficult for ICAPSA to directly show its advantages visually. Therefore, in **Table 4**, the values of PSNR and FSIM obtained in thresholding segmentation process are recorded too. As the number of thresholds gradually increased from 2 to 5 in **Table 4**, the distribution of thresholds in the range [0 255] was more balanced. In combination with the visual effects of **Figure 3**, the effectiveness of ICAPSA in plant image thresholding is illustrated from the data level. In addition, more detailed data comparison will be given in Section 4.3.

### 4.3 Comparison and analysis of similar algorithms

The visual and segmentation data analysis of ICAPSA alone cannot fully demonstrate its consistently reliable performance. To demonstrate the effects of ICAPSA more fully, we refer in this paper to the evaluation methods used in relevant comparative literature. We take PSNR value as the measurement standard, and compare it with the original CAPSA, FABC, DCOA, MWOA and ISBO. On the basis of keeping the original parameters of FABC, DCOA, MWOA and ISBO, the results are provided in **Table 5**. From the data comparison in **Table 5**, the effect of ICAPSA is visibly superior to the original CAPSA. The most obvious improvement occurred at Citrus Bacterial Canker image when the threshold number was 2, the value of PSNR increased by 6.0525, a proportional increase of 39.7%. In the worst case is at Powdery Mildew image, when the threshold was 2, the PSNR value also increased by 0.0142, with an increase of 0.07%. But overall, ICAPSA was proved about 10.6% higher than the original CAPSA. Compared with the experimental data of FABC, the effect of ICAPSA is also better than that of FABC. In the Citrus Bactrial Canker image, when the threshold is 2, the effect improvement is the largest, with an increase of about 91.3%. In general, the effect of ICAPSA is 12.5% higher than that of FABC on average. Compared with experimental results of DCOA, similarly, ICAPSA’s segmentation effect is slightly higher, with an average increase of 11.7%.

**Table 5:** Thresholding results with different segmentation algorithms.

Image	NT	PSNR					
		ICAPSA	CAPSA	FABC	DCOA	MWOA	ISBO
**Leaf01**	2	**16.2911**	16.1719	16.2788	15.9541	16.1141	16.2897
	3	**22.5087**	16.5023	16.5314	16.5394	16.7720	16.6751
	4	**25.7779**	21.9052	16.6181	16.7702	24.6964	17.5584
	5	**26.8305**	23.7037	16.8697	17.1991	26.2811	26.1164
**Apple Brown Spot**	2	**18.6521**	17.6643	18.5825	16.2137	17.2934	18.0229
	3	**21.2129**	20.0332	20.1522	20.9419	20.7366	20.5594
	4	**22.7506**	21.1680	21.9783	21.6583	21.8136	21.9044
	5	**23.9403**	22.8429	23.6587	23.3634	23.2905	23.2198
**Citrus Bacterial Canker**	2	**21.3084**	15.2559	11.1408	20.8239	13.7563	20.0875
	3	**24.4853**	21.1620	24.0375	22.6063	23.0258	23.0702
	4	**26.0432**	23.8553	25.4951	24.8464	24.1417	25.2638
	5	**27.1299**	25.6433	26.5732	26.4201	25.5010	25.7739
**Black Spot**	2	**21.5449**	18.6999	20.9316	18.8026	15.6189	20.2820
	3	**23.7496**	20.0288	22.1855	21.1415	21.0457	21.3856
	4	**25.1650**	23.7635	23.7159	24.2592	23.4749	23.6836
	5	**26.2449**	24.4805	24.6354	24.4448	24.9746	24.3882
**Bacterial keratosis of cucumber**	2	**19.2370**	17.3167	19.1072	17.5003	18.9013	19.1536
	3	**21.8199**	20.2393	20.9140	20.8431	19.9737	20.0132
	4	**23.6029**	22.0439	22.7280	22.8016	22.6521	22.1477
	5	**24.6088**	23.6152	23.4007	23.7169	23.4316	23.0604
**Powdery Mildew**	2	**20.0592**	20.0450	19.8182	17.4870	19.7587	19.3961
	3	**21.8992**	20.9081	21.4543	20.7328	21.3120	21.2948
	4	23.2750	22.1054	23.1606	22.0906	21.6392	**23.5397**
	5	**24.9361**	23.4687	24.3110	23.7426	22.3853	24.6649

Compared with the experimental data of MWOA, it is found that the segmentation effect of ICAPSA is generally slightly higher than that of MWOA, with an average improvement of 10%. Finally, from the comparison results with ISBO, except at Powdery Mildew image, when the threshold is 4, the effect of ICAPSA is lower than that of ISBO, and the PSNR difference is only 0.2647. From the overall analysis of the results, ICAPSA was about 7.5% higher than ISBO. From the analysis of the above results, it can be concluded that ICAPSA has better effects in plant image segmentation.

## 5 Conclusion

To improve the accuracy and effects of plant image segmentation, this paper combines and improves the traditional thresholding image segmentation by improving the CAPSA. It uses Tent chaotic map sequence and Opposite-based learning to improve the quality of the initial population and the ability of global optimization in ICAPSA. To avoid the problem of local optimization, Levy Flight disturbance strategy is introduced to make the algorithm mutate when updating the position, so as to balance the global optimization and local exploitation of ICAPSA. Finally, the Kapur entropy is used as the objective function to segment the plant images. The results are compared with CAPSA, FABC, DCOA, MWOA and ISBO. From these results, the improved CAPSA (ICAPSA) demonstrates superior segmentation effects in the field of plant image segmentation.

## Data availability statement

The original contributions presented in the study are included in the article/supplementary material. Further inquiries can be directed to the corresponding author.

## Author contributions

All authors listed have made a substantial, direct, and intellectual contribution to the work and approved it for publication.
